# Subclinical epileptiform activity and sleep disturbances in Alzheimer's disease

**DOI:** 10.1002/brb3.3306

**Published:** 2023-11-10

**Authors:** Astrid Devulder, Jaiver Macea, Alexandros Kalkanis, François‐Laurent De Winter, Mathieu Vandenbulcke, Rik Vandenberghe, Dries Testelmans, Maarten J. A. Van Den Bossche, Wim Van Paesschen

**Affiliations:** ^1^ Laboratory for Epilepsy Research, KU Leuven and Department of Neurology University Hospitals Leuven Leuven Belgium; ^2^ Laboratory of Respiratory Diseases and Thoracic Surgery (BREATHE), KU Leuven and Department of Pulmonary Diseases University Hospitals Leuven Leuven Belgium; ^3^ Division of Neuropsychiatry, Department of Neurosciences, Leuven Brain Institute, KU Leuven and Department of Geriatric Psychiatry University Psychiatric Center (UPC) KU Leuven Leuven Belgium; ^4^ Laboratory for Cognitive Neurology, KU Leuven and Department of Neurology University Hospitals Leuven Leuven Belgium

**Keywords:** Alzheimer's disease, epileptiform activity, obstructive sleep apnea, REM sleep, sleep

## Abstract

**Introduction:**

Subclinical epileptiform activity (SEA) and sleep disturbances are frequent in Alzheimer's disease (AD). Both have an important relation to cognition and potential therapeutic implications. We aimed to study a possible relationship between SEA and sleep disturbances in AD.

**Methods:**

In this cross‐sectional study, we performed a 24‐h ambulatory EEG and polysomnography in 48 AD patients without diagnosis of epilepsy and 34 control subjects.

**Results:**

SEA, mainly detected in frontotemporal brain regions during N2 with a median of three spikes/night [IQR1–17], was three times more prevalent in AD. AD patients had lower sleep efficacy, longer wake after sleep onset, more awakenings, more N1%, less REM sleep and a higher apnea‐hypopnea index (AHI) and oxygen desaturation index (ODI). Sleep was not different between AD subgroup with SEA (AD‐Epi+) and without SEA (AD‐Epi–); however, compared to controls, REM% was decreased and AHI and ODI were increased in the AD‐Epi+ subgroup.

**Discussion:**

Decreased REM sleep and more severe sleep‐disordered breathing might be related to SEA in AD. These results could have diagnostic and therapeutic implications and warrant further study at the intersection between sleep and epileptiform activity in AD.

## 1 BACKGROUND

There is an increased prevalence of seizures and subclinical epileptiform activity (SEA) in Alzheimer's Disease (AD), even early in the disease course (Amatniek et al., 2006; Friedman et al., 2012; Horvath et al., 2021; Lam et al., 2020; Vossel et al., 2017; Vossel et al., 2013). AD patients with SEA have faster cognitive decline (Horvath et al., 2021; Vossel et al., 2016), and treatment with antiseizure medication (ASM) had a positive effect on executive functions and spatial memory (Vossel et al., 2021). For these reasons, it is crucial to identify AD patients with an increased risk for SEA. However, this is not easy in the clinical practice: prolonged EEG recordings are labor‐ and cost‐intensive, patients with AD often do not tolerate long‐ and overnight EEG recordings, and mesial temporal lobe epileptiform activity often lacks correlates on scalp EEG or is not associated with clinical symptoms (Lam et al., 2016; Lam et al., 2017; Lieb et al., 1976).

Sleep is often disrupted in AD, and these patients have a higher prevalence of obstructive sleep apnea syndrome (OSAS) (Hoch et al., 1986; Peter‐Derex et al., 2015; Reynolds et al., 1985; Romanella et al., 2021). Like epileptiform activity, sleep disturbances may occur early in the disease course and are associated with decreased cognitive functioning (D'Rozario et al., 2020; Holth et al., 2017; Lucey et al., 2021), and OSAS treatment may slow cognitive decline in AD (Ancoli‐Israel et al., 2008; Liguori et al., 2021; Troussiere et al., 2014). Moreover, epileptiform activity and sleep are two entities with an important relation with cognition and have a complex bidirectional relationship (Derry & Duncan, 2013; Manni & Terzaghi, 2010; Matos et al., 2013; van Golde et al., 2011). NREM sleep facilitates epileptic activity (Nobili et al., 2022), and ictal and interictal activity have an arousing effect on sleep (Peter‐Derex et al., 2020). Reduced REM sleep has been reported as a potential biomarker for epilepsy (Sadak et al., 2022). OSAS is more prevalent in epilepsy (Lin et al., 2017) and OSAS treatment improves seizure control in patients with epilepsy (Lin et al., 2017; Pornsriniyom et al., 2014).

We hypothesized that there is an interaction between the presence of epileptiform activity and sleep disturbances in AD. To study this interaction, we performed 24‐h EEG recordings with concomitant polysomnography (PSG). This combined EEG‐PSG set‐up allowed assessment of respiratory parameters and reliable REM sleep scoring, which can be challenging on scalp EEG alone.

## 2 METHODS

### 2.1 Standard protocol approval, registration, and patient consent

All prospective study procedures were performed under study protocol, approved by the Ethical Committee of University Hospitals Leuven (clinical.trials.gov NCT03617497). Written informed consent was obtained from all research participants or their caregivers.

### 2.2 Patient selection

Research participants between 55 and 85 years old were prospectively recruited between September 2019 and December 2022. Patients with AD were selected from the memory clinic of University Hospitals Leuven. They had either probable clinical AD or probable AD dementia with evidence of the AD pathophysiological process according to the National Institute on Aging‐Alzheimer's Association diagnostic guidelines (Albert et al., 2011; McKhann et al., 2011) (Appendix A). All control subjects were healthy volunteers who were free of subjective cognitive decline and had a Mini Mental State Examination (MMSE) score ≥ 27/30, a Clinical Dementia Rating scale (CDR) score of 0, and normal neurological examination. Exclusion criteria for both patients and control subjects were as follows: (1) history of seizures or diagnosis of epilepsy; (2) evidence of any other clinically significant neurological disorder; (3) severe comorbidity that would influence sleep significantly, including severe heart, lung, kidney, or liver disease; (4) current diagnosis of alcohol abuse; (5) OSAS treated with Continuous Positive Airway Pressure (CPAP); (6) the subject was started on or had changes in benzodiazepines dose in the last 4 weeks.

### 2.3 Clinical measures

For each person with AD, we registered their age, duration of symptoms, body mass index (BMI), medication use, comorbidities, caffeine consumption, and alcohol and tobacco use. The patient completed the MMSE (Folstein et al., 1975) as cognitive screening test and we performed a clinical interview with Pittsburg Sleep Quality Index (PSQI) (Mollayeva et al., 2016), Epworth Sleepiness Scale (ESS) (Johns, 1991; Scharf, 2022), Mayo fluctuation scale (Ferman et al., 2004), Cornell scale for depression in dementia (Alexopoulos et al., 1988; Park & Cho, 2022), CDR (Juva et al., 1995), and Reutens Questionnaire for clinical seizure diagnosis (Reutens et al., 1992). The same clinical variables were collected in control subjects except for the duration of symptoms and the Mayo fluctuation scale.

### 2.4 EEG and PSG recordings

Twenty‐four‐hour ambulatory EEG and PSG recordings were acquired with Medatec Brainwalker 3 device and software (Medical Data Technology, Braine‐le‐Château, Belgium), using a sampling rate of 200 Hz. The EEG and PSG recordings occurred at home for all participants. Scalp electrodes were placed using the International 10–20 system with additional lower temporal electrodes (F9, F10, T9, T10, P9, P10) without P3 and P4, and Cz was used as ground. We placed two additional chin electrodes (EMG 1 and 2) and two electrodes next to the eyes (EOG 1 and EOG 2) to measure sleep. Additional sensors were nasal flow (NAF2P), thoracic and abdominal resistance bands, finger pulse oximetry, electrocardiogram (ECG) derivation Eindhoven I, and electromyogram (EMG) of one leg. Finally, a camera with audio recording was installed in the patient's bedroom for nighttime recording.

### 2.5 EEG review and annotation

EEG's were visually reviewed and annotated by the first author (A.D.) and discussed with a certified neurologist (W.V.P.). All seizures, interictal epileptiform discharges (IED), and episodes of temporal rhythmic delta activity (TIRDA) were annotated. Persyst 14 software (Persyst, CA, USA) was additionally used for automatic spikes detections, which were visually reviewed by first author (A.D.). All annotations were blindly shown to two certified neurologists (W.V.P. and J.M.), which scored them according to the International Federation for Clinical Neurophysiology criteria (Kural et al., 2020). In case of disagreement, the readers reached a consensus after open discussion. IED location was visually determined based on the electrode with the highest amplitude in common referential or phase opposition in bipolar montages. TIRDA was defined as short trains, between 1 and 10 s, of intermittent rhythmic delta activity (1 and 4 Hz, 50–100 μV) in the temporal chains Brigo, 2011, Normand et al., 1995; Reiher et al., 1989). The EEG was defined as epileptiform if one or more of the following occurred: seizure, IED, or TIRDA (Di Gennaro et al., 2003; Gambardella et al., 1995; Lam et al., 2020).

### 2.6 PSG review and annotation

Two groups of readers visually reviewed all PSG and scored them according to the American Academy of Sleep Medicine (AASM) scoring rules version 2.6 (Berry et al., 2020). The first group was composed by an experienced sleep technologist (P.V.H. or I.M.) and the second group by a certified somnologist (A.K. or D.T.) or the first author. In case of disagreement, consensus was reached after open discussion. Electrodes P9 and P10 were used as reference electrodes instead of A1 and A2. Apneas were scored based on the flow signal on NAF2P. The apnea‐hypopnea index (AHI) was defined as the total number of apneas and hypopneas per hour of sleep. The oxygen desaturation index (ODI) was defined as the number of desaturations ≥3% per hour of sleep. OSAS was defined as the presence of an obstructive apnea‐hypopnea index (OAHI) ≥ 5/h (mild ≥ 5 OAHI < 15; moderate ≥15 OAHI < 30; severe OAHI ≥ 30).

### 2.7 Statistical analysis

Statistical analysis was performed in R software version 4.1.2. To compare clinical parameters, OSAS and IED prevalence, we used Student's *t*‐test, Wilcoxon rank‐sum test, chi‐squared test, or Fisher's exact test as appropriate. We used multiple linear regression analysis using age, AHI on total sleep time (TST), and use of sleep medication, antidepressive, or antipsychotic drugs to compare sleep between AD and control group and between AD patients with SEA (AD‐Epi+ subgroup) and without SEA (AD‐Epi– subgroup). Bonferroni correction for multiple comparisons (*p*‐value ≤ .025) was applied when comparing AD‐Epi+ subgroup and AD‐Epi– subgroup with the control group. Statistical analysis of EEG data was limited to time in bed (TIB).

## 3 RESULTS

We included 48 patients with AD. Seven were excluded due to technical failure of the device (*n* = 3) or behavioral problems (*n* = 4). Thirty‐four control subjects were included, but four were excluded from the analysis due to intolerance to the EEG (*n* = 2) equipment or excessive use of psychotropic drugs or alcohol above the limit of three daily units that could influence sleep (*n* = 2). For PSG analysis, three additional AD subjects and one control subject were excluded due to insufficient signal quality to obtain a reliable scoring of respiratory events.

### 3.1 Demographics (Table 1)

The mean age of AD patients was 4 years higher than the control group. Three AD patients took a benzodiazepine (7%). Thirteen AD patients (32%) and one control participant (3%) were on antidepressive or antipsychotic medication. Although no formal diagnosis of epilepsy was made at moment of study participation, three patients with AD had a positive Reutens Questionnaire for clinical seizure diagnosis: one patient had episodes of confusional awakenings during sleep and episodes where she became pallor, hyperventilated and fainted; the second patient experienced the past year three episodes of staring with partial loss of the postural tone; and the third patient had experienced three episodes of staring with automatisms and dystonia of the right arm. No patient encountered an episode during study participation. ESS and PSQI scores were similar in AD and control groups. Twelve AD patients had an MMSE ≥27 and were further classified as having mild cognitive impairment (MCI). Clinical characteristics are summarized in Table 1. Biomarkers were available for 24 AD patients (Appendix A).

**TABLE 1:** Clinical parameters of study population.

	Controls (*n* = 30)	AD (*n* = 41)	*p*‐Value
Number of male (%)	11 (37%)	22 (54%)	N.S.
Number of patients with MCI (%)	N.A.	12 (29%)	N.A.
Median duration of symptoms in years [IQR]	N.A.	3 [2–5]	N.A.
Mean age in years (SD)	71 (7)	75 (7)	.009*
Mean BMI in kg/m2 (SD)	24.70 (3.91)	23.43 (3.43)	N.S.
Median units ethyl/day [IQR]	0 [0–1]	0 [0−1]	N.S.
Median units caffeine consumption day [IQR]	3 [2–5]	3 [2–5]	N.S.
Number of participants who smoke (%)	3 (10%)	5 (12%)	N.S.
Number of participants who took benzodiazepine, antidepressive or antipsychotic drug (%)	1(3%)	16 (39%)	.001*
Median MMSE [IQR]	29 [28–30]	20 [15–27]	<.001*
Median CDR [IQR]	NA	1 [0.5 −2.0]	N.A.
Median ESS [IQR]	4 [2–7]	5 [2–8]	N.S.
Median PSQI [IQR]	4 [2–6]	3 [2 −5]	N.S.
Median Cornell [IQR]	NA	5 [2–10]	N.A.
Median Mayo [IQR]	NA	1 [0−2]	N.A.
Number of participants with Positive Reutens Questionnaire (%)	0 (0%)	3 (7%)	N.S.

Abbreviations: BMI = body mass index; CDR = Clinical Dementia Rating Scale; ESS = Epworth Sleepiness Scale; IQR = interquartile range; MCI = mild cognitive impairment; MMSE = Mini Mental State Examination; N.A. = not applicable; N.S. = not significant; PSQI = Pittsburg Sleep Quality Index; SD = standard deviation.

*Significant *p*‐values (≤.05).

**TABLE 2:** EEG parameters controls versus AD.

	Controls (*n* = 30)	AD (*n* = 41)	*p*‐Value
**Overal epileptiform EEG**			
Number of participants with overall epileptiform EEG (%)	4 (13%)	14 (34%)	.086
Number of participants with spikes (%)	4 (13%)	13 (32%)	N.S.
Number of participants with TIRDA (%)	0 (0%)	1 (2%)	N.S.
Number of MCI participants with epileptiform EEG (%)	N.A.	6 (50%)	N.A.
Number of dementia patients with epileptiform EEG (%)	N.A.	8 (28%)	N.A.
**Quantification of spikes**			
Total number of spikes during TIB	12	234	N.A.
Median number of spikes during TIB [IQR] in patients with epileptiform EEG	1 [1–3]	3 [1–17]	N.S.
**Spatial distribution of spikes**			
Main electrodes involved	F7 (50%) > T9 (42%) > F9 (8%)	T9 (22%) > F3 (16%) & F9 (16%)	N.A.
**Temporal distribution of spikes (% normalized per participant)**			
Median % of spikes in N1 [IQR]	3% [0%−6%]	0% [0%−0%]	N.S.
Median % of spikes in N2 [IQR]	64% [58%−91%]	47% [29%−96%]	N.S.
Median % of spikes in N3 [IQR]	26% [6%−31%]	7% [0%−21%]	N.S.
Median % of spikes in REM [IQR]	0% [0%−0%]	0% [0%–0%]	N.S.
Median % of spikes in Awake [IQR]	0% [0%−0%]	4% [0%–31%]	.029*

Abbreviations: IQR = interquartile range; MCI = mild cognitive impairment; N.A. = not applicable; N.S. = not significant; TIB = time in bed; TIRDA = temporal intermittent rhythmic delta activity.

*Significant *p*‐values (≤.05).

**TABLE 3:** Polysomnography parameters controls versus AD.

Parameter	Controls (*n* = 29)	AD (*n* = 38)	*p*‐Value
**Respiration**			
ODI on TST median [IQR]†	9 [5−14]	18 [10–36]	.005*
AHI median [IQR]^†^	7 [3–15]	16 [8–28]	.021*
OAHI median [IQR]	6 [1–14]	13 [5−23]	N.S.
Mean saturation during TST (SD)	94% (1%)	94% (2%)	N.S.
OSAS *n* (%)	15/29 (52%)	28/38 (74%)	N.S.
**Sleep**			
Total time in REM mean (SD)	98 min (28 min)	66 min (36 min)	<.001*
REM% mean (SD)	26% (6%)	20% (10%)	.006*
WASO median [IQR]	65 min [28–105 min]	137 min [78−162 min]	.001*
SE% mean (SD)	78% (9%)	66% (15%)	.002*
N1% median [IQR]^‡^	3% [2%−4%]	6% [3%–12%]	.012*
Awakening Index on TST median [IQR]^‡^	4 [3–5]	6 [5–10]	.013*
AI on TST median [IQR]	17 [11–19]	23 [15–34]	.052
Total time in N1 median [IQR]^‡^	13 min [8–16 min]	21 min [10–39 min]	.057
TST mean (SD)	379 min (68 min)	345 min (92 min)	.081
TIB median [IQR]	482 min [453–533 min]	544 min [491–573 min]	.087
Sleep latency median [IQR]	20 min [11–29 min]	25 min [12–40 min]	N.S.
REM latency median [IQR]^‡^	58 min [42–85 min]	70 min [50–147 min]	N.S.
N2% mean (SD)	46% (11%)	52% (15%)	N.S.
Total time in N2 median [IQR]	172 min [143–197 min]	166 min [113–221 min]	N.S.
N3% median [IQR]	25% [17%–32%]	19% [11%–28%]	N.S.
Total time in N3 median [IQR]	91 min [70–122 min]	63 min [30–106 min]	N.S.

Abbreviations: AHI = apnea‐hypopnea index; AI = arousal index; IQR = interquartile range; N.S. = not significant; OAHI = obstructive apnea‐hypopnea index; ODI = oxygen desaturation index; OSAS = obstructive sleep apnea syndrome; SD = standard deviation; SE = sleep efficacy; TIB = time in bed; TST = total sleep time; WASO = wake after sleep onset.

Correction for age, medication and AHI on TST. Except for AHI, OAHI, and ODI on TST only for age and medication.

*Significant *p*‐values (≤.05).

^†^ Original values are represented, but statistical testing on square‐root‐transformed data.

^‡^ Original values are represented, but statistical testing on log‐transformed data.

**TABLE 4:** Polysomnography parameters controls versus AD‐Epi ± and AD‐Epi– subgroups.

Parameter	Controls (*n* = 29)	AD‐Epi+ (*n* = 13)	AD‐Epi– (*n* = 25)	*p*‐Value AD‐Epi+ vs. control^§^	*p*‐Value AD‐Epi– vs. control^§^	*p*‐Value AD‐Epi+ vs. AD‐Epi–
**Respiration**						
ODI on TST median [IQR]^†^	9 [5−14]	22 [10−38]	18 [11–36]	.002*	.056	N.S.
AHI median [IQR]^†^	7 [3–15]	18 [8−27]	16 [9–28]	.012*	N.S.	N.S.
OAHI median [IQR]^†^	6 [1–14]	18 [7–23]	13 [5–22]	N.S.	N.S.	N.S.
Mean saturation during TST (SD)	94% (1%)	94% (2%)	94% (2%)	N.S.	N.S.	N.S.
OSAS *n* (%)	15 (52%)	10 (77%)	18 (72%)	N.S.	N.S.	N.S.
**Sleep**						
REM% mean (SD)	26% (6%)	17% (11%)	21% (9%)	.006*	.034	N.S.
Total time in REM sleep mean (SD)	98 min (28 min)	55 min (35 min)	73 min (33 min)	< .001*	.009*	N.S.
WASO median [IQR]	65 min [28–105 min]	142 min [98–162 min]	136 min [42–162 min]	.004*	.005*	N.S.
SE% mean (SD)	78% (9%)	65% (14%)	67% (16%)	.007*	.008*	N.S.
N1% median [IQR]^‡^	3% [2%–4%]	4% [3%–8%]	8% [4%–15%]	N.S.	.003*	N.S.
Total time in N1 median [IQR]^‡^	13 min [8–16 min]	13 min [9–27 min]	27 min [13–42 min]	N.S.	.010*	0.098
Awakening Index median [IQR]^‡^	4 [3–5]	6 [5–9]	6 [4–11]	N.S.	.012*	N.S.
AI median [IQR]	16 [11–19]	22 [15–35]	23 [16–31]	N.S.	.063	N.S.
TST mean (SD)	379 min (68 min)	322 min (101 min)	358 min (86 min)	.037	N.S.	N.S.
TIB median [IQR]	482 min [453–533 min]	523 min [481–562 min]	544 min [210–577 min]	N.S.	.050	N.S.
Sleep latency median [IQR]	20 min [11–29 min]	24 min [14–39 min]	26 min [13–42 min]	N.S.	N.S.	N.S.
REM latency median [IQR]^‡^	58 min [42–85 min]	60 min [45–80 min]	80 min [53–159 min]	N.S.	.038	N.S.
N2% mean (SD)	46% (11%)	51% (17%)	50% (11%)	N.S.	N.S.	N.S.
Total time in N2 mean (SD)	175 min (53 min)	163 min (77 min)	177 min (56 min)	N.S.	N.S.	N.S.
N3% median [IQR]	25% [17%−32%]	23% [11%–35%]	19% [14%−24%]	N.S.	N.S.	N.S.
Total time in N3 median [IQR]	91 min [70–122 min]	62 min [25–122 min]	63 min [39–93 min]	N.S.	N.S.	N.S.

Abbreviations: AHI = apnea‐hypopnea index; AI = arousal index; IQR = interquartile range; N.S. = not significant; OAHI = obstructive apnea‐hypopnea index; ODI = oxygen desaturation index; OSAS = obstructive sleep apnea syndrome; SD = standard deviation; SE = sleep efficacy; TIB = time in bed; TST = total sleep time; WASO = wake after sleep onset.

Correction for age, medication and AHI. Except for AHI, OAHI, and ODI on TST only for age and medication.

*Significant *p*‐values.

^†^ Original values are represented, but statistical testing on square‐root‐transformed data.

^‡^ Original values are represented, but statistical testing on log‐transformed data.

^§^ Significant *p*‐value ≤ .025 according to Bonferroni correction for multiple comparisons.

### 3.2 EEG parameters (Table 2)

Fourteen of 41 patients with AD (34%) had an interictal epileptiform EEG, with a higher prevalence in the MCI (50%) versus the dementia subgroup (28%). The prevalence was almost three times higher than in controls, where 4 of 30 participants (13%) had one or more spikes on scalp EEG (*p*‐value = .086). Of 14 AD patients with an epileptiform EEG, 13 had one or more spikes, and 1 had TIRDA exclusively during wakefulness. Two hundred and thirty‐four spikes were marked on scalp EEG in the AD group versus 12 spikes in the control group. In subjects with spikes, the median was three [IQR 1−17] in the AD group and one [IQR 1–3] in the control group, both measured during TIB (*p*‐value = .358). In the AD group, nine patients had spikes exclusively in frontotemporal brain regions, and four patients had them in other lobes. Main electrodes involved were T9 (22%), followed by F3 (16%) and F9 (16%). All control subjects with spikes had epileptiform abnormalities only in the left frontotemporal region, with F7 (50%), T9 (42%), and F9 (8%) as the main involved electrodes. In AD patients, IEDs were present during sleep and wakefulness through TIB, whereas in control subjects, they occurred exclusively during sleep. In both groups, epileptiform spikes were most frequent during sleep stage N2. Median spike temporal distribution was significantly different during wakefulness between AD (4% [IQR 0%–31%]) and controls (0% [IQR 0%–0%]) (*p*‐value = .029). Clinical parameters between the AD‐Epi+ and AD‐Epi– were comparable. One AD‐Epi– subgroup participant had a positive Reutens Questionnaire for clinical seizure diagnosis, but no epileptiform discharges were identified on scalp EEG (Appendix B).

### 3.3 PSG parameters

#### 3.3.1 AD versus controls (Table 3)

OSAS was present in 74% of AD (mild in 43%, moderate in 36%, and severe in 21%) and 52% of control subjects (*p*‐value = .110) (67% mild and 33% moderate). The total AHI was significantly higher in the AD group (median AHI on TST 16/h vs. 7/h, *p*‐value = .021). The median ODI on TST in the AD group was 18/h, twice as high as in the control group (*p*‐value = .005). AD patients had 6% less REM sleep (*p*‐value = .006) or spent an average of 33 min less in REM sleep (*p*‐value < .001), and the median wake after sleep onset (WASO) was 72 min longer compared to controls (*p*‐value = .001). The AD group's mean sleep efficacy (SE) was 12% lower (*p*‐value = .002), since AD patients tended to have a longer TIB (*p*‐value = .087) and shorter TST (*p*‐value = .081). The median N1% was 3% higher in the AD group versus controls (*p*‐value = .012). AD patients had a median awakening index (AwI) of 6/h, compared to 4/h in the control group (*p*‐value = .013). The AD group's arousal index (AI) was 23/h, not significantly different from 17/h in the control group (*p*‐value = .052).

#### 3.3.2 PSG parameters in AD‐Epi+, AD‐Epi–, and controls (Table 4)

We performed PSG analysis in AD‐Epi+ and AD‐Epi– subgroups. There was no difference in the prevalence of OSAS or OAHI between AD‐Epi+, AD‐Epi–, and controls (*p*‐value = N.S.). However, compared to controls, the AHI (*p*‐value = .012 for AD‐Epi+ vs. controls) and ODI (*p*‐value = .002 for AD‐Epi+ vs. controls) were significantly higher in the AD‐Epi+ subgroup. The total REM sleep time was significantly decreased in both AD‐Epi+ and AD‐Epi– subgroups compared to controls (*p*‐value < .001 for AD‐Epi+ vs. controls; *p*‐value = .009 for AD‐Epi– vs. controls). REM% was decreased in the AD‐Epi+ subgroup compared to controls (*p*‐value = .006 fAD‐Epi+ vs. controls). The mean SE was lower in both AD subgroups (*p*‐value = .007 for AD‐Epi+ vs. controls; *p*‐value = .008 for AD‐Epi– vs. controls), and the median WASO was longer (*p*‐value = .004 for AD‐Epi+ vs. controls; *p*‐value = .005 for AD‐Epi– vs. controls) in both subgroups compared to controls. The AwI was significantly higher in the AD‐Epi– subgroup (*p*‐value = .012 for AD‐Epi– vs. controls). The AD‐Epi– subgroup spent more absolute time in N1 sleep stage (*p*‐value = .010 for AD‐Epi– vs. controls) and more relative time in N1 sleep stage than controls (*p*‐value = .003 for AD‐Epi– vs. controls). There were no significant differences in sleep measurements between the AD‐Epi+ and AD‐Epi– subgroups (*p*‐value = N.S.).

## 4 DISCUSSION

We performed a cross‐sectional study to evaluate the presence of SEA and sleep alterations in AD. Our results show that patients with AD have (1) a three times higher prevalence of SEA, (2) a decrease in total time in REM sleep and %REM sleep, particularly in AD‐Epi+, (3) a longer WASO, (4) a decreased SE, (5) more N1 sleep (6) a higher AwI, and (7) a higher AHI and ODI on TST, particularly in AD‐Epi+ subgroup.

In this cross‐sectional study with prolonged EEG recording in AD patients without a formal diagnosis of epilepsy, we did not capture seizures. However, SEA was present in 34%, almost three times more than in the control population (13%), congruent with previous work (Horvath et al., 2021; Vossel et al., 2016). Although 34% is a high prevalence, the median frequency of three spikes per night is rather low. Epileptiform discharges were mainly detected in frontotemporal brain regions, reflecting their possible involvement in AD pathogenesis (Vossel et al., 2017) and mainly occurred during stage N2, which is known to promote epileptiform activity. In our AD cohort, 22% of spikes were during wakefulness, compared with only 8% and 9.9% in previous work by Horvath et al. (2021) and Vossel et al. (2016). There is no gold standard regarding when and how to search for epileptic activity in AD. We performed a prolonged overnight EEG recording, which is more sensitive than standard routine EEG recordings (Horvath et al., 2017; Vossel et al., 2013), but still has limited sensitivity in detecting mesial temporal lobe epileptiform activity as is proven by combined scalp‐intracranial recordings (Abou Jaoude et al., 2022; Lam et al., 2017; Lieb et al., 1976). In fact, one patient of our AD‐Epi– subgroup had a positive Reutens Questionnaire for clinical seizure diagnosis, although we did not detect any epileptiform abnormalities on scalp EEG. Thus, some patients in the AD‐Epi– subgroup may actually belong to the AD‐Epi+ subgroup. Intracranial and prolonged video‐EEG recordings are cost‐ and labor‐intensive and often not tolerated in a more advanced disease stage. The extrapolation of deep learning algorithms for detecting epileptiform activity on scalp EEG (Abou Jaoude et al., 2022) will be of great value in searching SEA biomarkers in AD.

Patients with AD in our study had a lower SE, more awakenings, more N1%, a longer WASO, and shorter REM%, confirming previous reports (D'Rozario et al., 2020; Zhang et al., 2022). Our cross‐sectional study focused on interactions between these sleep disorders and SEA in AD. Although we did not find statistically significant differences in sleep parameters between the AD‐Epi+ and AD‐Epi– subgroups, our findings on decreased REM sleep in AD warrant further discussion. REM sleep is the least permissive state for epileptiform activity and is reduced in patients with epilepsy (Bazil et al., 2000, Sadak et al., 2022; Yeh et al., 2022), including patients with refractory epilepsy, compared to those with controlled epilepsy (Yeh et al., 2021). Sadak et al. (2022) reported that the diagnosis of epilepsy based on REM% prediction models performed similar to prediction based on the presence of IED on video‐EEG and suggested that reduced REM sleep could be a biomarker for epilepsy. We observed an average REM sleep duration in healthy controls of 98 min, which was significantly reduced with 33% to 66 min in AD. More specifically compared to controls, the mean total REM sleep duration dropped with 44% in the AD‐Epi+ subgroup and 26% in AD‐Epi– subgroup and REM% dropped with 35% in AD‐Epi+ subgroup where this was only 19% in AD‐Epi– subgroup. We might speculate that REM sleep in AD might be shorter because of an underlying undiagnosed epilepsy. Intracranial recordings and larger sample size studies are necessary to prove this hypothesis. The reliability of specific sleep staging with wearable devices is still quite low (Miller et al., 2022) and requires improvements before sleep parameters, like decreased REM%, could become a noninvasive, easy‐to‐measure biomarker to identify those patients at risk for/with SEA. The higher AwI and more time spent in N1 sleep was significantly higher in AD‐Epi– subgroup compared to controls. We expected it would be higher in AD‐Epi+ subgroup since nighttime temporal lobe seizures are associated with higher N1% (Bazil et al., 2000) and scalp‐negative temporal lobe seizures, which occur during sleep, are often associated with awakenings (Lam et al., 2016). However, these values might be influenced by the relatively small and unequal sample sizes, where AD‐Epi– subgroup was almost twice as large as the AD‐Epi+ subgroup.

AD patients have a five times higher chance of having OSAS than cognitively unimpaired older adults (Emamian et al., 2016). The prevalence of OSAS was not significantly higher in our AD cohort; however, six AD patients had severe OSAS where this was absent in the control sample, which is reflected in higher ODI and AHI values. These observations were not reflected in the questionnaires, where ESS and PSQI scores were similar across patients and controls, and no subjects reported symptoms of severe daytime sleepiness or poor sleep quality. These findings possibly reflect the problem of underreporting of sleep problems in the elderly (Gordon et al., 2022) and the low reliability of questionnaires in cognitively impaired patients. Animal and human studies provide accumulating evidence for an interaction between OSAS and AD pathogenesis by effects of sleep disruption and intermittent hypoxia on amyloid and tau dynamics in cerebrospinal fluid and brain tissue (Bu et al., 2015; Holth et al., 2017; Holth et al., 2019; Ju et al., 2016; Liguori et al., 2017; Liguori et al., 2019; Motamedi et al., 2018; Osorio et al., 2014; Osorio et al., 2015; Spira et al., 2014). CPAP treatment may positively affect cognitive functions in AD (Ancoli‐Israel et al., 2008; Liguori et al., 2021; Troussiere et al., 2014). Furthermore, there is a complex interaction between OSAS and epilepsy. OSAS is more prevalent in patients with epilepsy, and CPAP treatment is associated with better seizure reduction and beneficial effects on interictal EEG (Lin et al., 2017; Pornsriniyom et al., 2014). Moreover, there is evidence that the hippocampus, prone to epileptiform activity, might play a role in breathing patterns (Harper et al., 1998). We were interested if SEA and sleep‐disordered breathing (SDB) were related in AD. There was no difference in prevalence of OSAS, AHI and ODI on TST between AD‐Epi+ and AD‐Epi– subgroups. The increased AHI and ODI on TST in AD‐Epi+ subgroup compared to controls supports a possible bidirectional relation between SDB and SEA in AD pathology, which can have diagnostic and therapeutic implications in the way of screening for SEA in AD patients with OSAS and vice‐versa.

Our results point toward a hypothesis of a complex interaction between SEA and sleep in AD. Further research should focus on an integrated approach to the role of SEA and sleep in AD to (1) better understand the role of SEA and sleep/SDB in AD pathogenesis; (2) find biomarkers for identifying those patients who are at risk for SEA; and (3) to find those patients in the heterogeneous AD population where antiseizure medication or CPAP treatment may have a significant positive benefit on their cognitive functions.

Our study has several limitations. First, our sample size was relatively small. More studies are needed before generalizing these results. Second, biomarker data were available for 16 AD patients, whereby another underlying pathology cannot be excluded. Third, our control population consisted of healthy volunteers without subjective cognitive complaints and MMSE scores between 27/30 and 30/30. No data were available for neuropsychological testing, neuroimaging, or biomarker status of our control sample. Therefore, we cannot rule out that some control participants had preclinical AD. Fourth, since two consecutive nights of PSG were difficult to achieve in the AD population, we recorded only one night of PSG. A first‐night effect could have influenced our results. Last, we focused only on sleep macrostructure. Focal epilepsy disrupts sleep spindles, which are essential for memory and learning (Schiller et al., 2022). Analyzing sleep microstructure could give more insight into the role of SEA and sleep in AD.

## 5 CONCLUSION

Our study showed a higher prevalence of SEA and sleep disturbances in AD. There were no significant differences in sleep macrostructure between AD patients with SEA and those without SEA on scalp EEG, probably influenced by the small sample size. However, REM sleep was most decreased in AD patients with SEA, which might constitute a potential biomarker for SEA in AD. The AHI and ODI on TST were higher in AD, specifically the subgroup with SEA. These findings suggest a complex interaction between sleep and epileptiform activity in AD. Further research, using concomitant intracranial recordings and wearables, should focus on an integrating approach to epilepsy and sleep in AD pathogenesis.

## AUTHOR CONTRIBUTIONS

**Astrid Devulder**: Data curation; analysis; methodology; writing—original draft; writing—review and editing. **Jaiver Macea**: Analysis; writing—review and editing. **Alexandros Kalkanis**: Analysis; writing—review and editing. **François‐Laurent De Winter**: Resources; writing—review and editing. **Mathieu Vandenbulcke**: Resources; writing—review and editing. **Rik Vandenberghe**: Resources; writing—review and editing. **Dries Testelmans**: Analysis; methodology; writing—review and editing. **Maarten J. A. Van Den Bossche**: Methodology; writing—review and editing. **Wim Van Paesschen**: Conceptualization; methodology; analysis; supervision; writing—original draft; writing—review and editing.

## CONFLICT OF INTEREST STATEMENT

The authors declare to have no conflicts of interest related to this study.

## CONSENT STATEMENT

All patients and control subject provided informed consent.

### FUNDING

This study was supported by Bijzonder Onderzoeksfonds (BOF) KU Leuven (Internal Funds KU Leuven) [C24/18/097].

### PEER REVIEW

The peer review history for this article is available at https://publons.com/publon/10.1002/brb3.3306.

## Supporting information

APPENDIX A: BIOMARKER DATA AND DISEASE STAGE OF AD PATIENTS
APPENDIX B: CLINICAL AND DEMOGRAPHICAL PARAMETERS AD‐EPI+ VERSUS AD‐EPI– SUBGROUP

## Data Availability

The data that support the findings of this study are available from the corresponding author upon reasonable request.

## References

[brb33306-bib-0001] Abou Jaoude, M. , Jacobs, C. S. , Sarkis, R. A. , Jing, J. , Pellerin, K. R. , Cole, A. J. , Cash, S. S. , Westover, M. B. , & Lam, A. D. (2022). Noninvasive detection of hippocampal epileptiform activity on scalp electroencephalogram noninvasive detection of hippocampal epileptiform activity on scalp electroencephalogram. JAMA Neurology, 79, 614. 10.1001/jamaneurol.2022.0888 35499837 PMC9062772

[brb33306-bib-0002] Albert, M. S. , Dekosky, S. T. , Dickson, D. , Dubois, B. , Feldman, H. H. , Fox, N. C. , Gamst, A. , Holtzman, D. M. , Jagust, W. J. , Petersen, R. C. , Snyder, P. J. , Carrillo, M. C. , Thies, B. , & Phelps, C. H. (2011). The diagnosis of mild cognitive impairment due to Alzheimer's disease: Recommendations from the National Institute on Aging‐Alzheimer's Association workgroups on diagnostic guidelines for Alzheimer's disease. Alzheimer's & Dementia, 7(3), 270–279. 10.1016/j.jalz.2011.03.008 PMC3312027 21514249

[brb33306-bib-0003] Alexopoulos, G. S. , Abrams, R. C. , Young, R. C. , & Shamoian, C. A. (1988). Cornell scale for depression in dementia. Biological Psychiatry, 23(3), 271–284. 10.1016/0006-3223(88)90038-8 3337862

[brb33306-bib-0004] Amatniek, J. C. , Hauser, W. A. , Delcastillo‐Castaneda, C. , Jacobs, D. M. , Marder, K. , Bell, K. , Albert, M. , Brandt, J. , & Stern, Y. (2006). Incidence and predictors of seizures in patients with Alzheimer's disease. Epilepsia, 47(5), 867–872. 10.1111/j.1528-1167.2006.00554.x 16686651

[brb33306-bib-0005] Ancoli‐Israel, S. , Palmer, B. W. , Cooke, J. R. , Corey‐Bloom, J. , Fiorentino, L. , Natarajan, L. , Liu, L. , Ayalon, L. , He, F. , & Loredo, J. S. (2008). Cognitive effects of treating obstructive sleep apnea in Alzheimer's disease: A randomized controlled study. Journal of the American Geriatrics Society, 56(11), 2076–2081. 10.1111/j.1532-5415.2008.01934.x 18795985 PMC2585146

[brb33306-bib-0006] Bazil, C. W. , Castro, L. H. M. , & Walczak, T. S. (2000). Reduction of rapid eye movement sleep by diurnal and nocturnal seizures in temporal lobe epilepsy. Archives of Neurology, 57(3), 363–368. 10.1001/archneur.57.3.363 10714662

[brb33306-bib-0007] Berry, R. B. , Quan, S. F. , & Abreu, A. R . (2020). The AASM manual for the scoring of sleep and associated events: Rules, terminology and technical specifications. Version 2.6. Westchester, IL: American Academy of Sleep Medicine.

[brb33306-bib-0008] Brigo, F. (2011). Intermittent rhythmic delta activity patterns. Epilepsy & Behavior, 20(2), 254–256. 10.1016/j.yebeh.2010.11.009 21276757

[brb33306-bib-0009] Bu, X.‐L. , Liu, Y.‐H. , Wang, Q.‐H. , Jiao, S.‐S. , Zeng, F. , Yao, X.‐Q. , Gao, D. , Chen, J. I.‐C. , & Wang, Y.‐J. (2015). Serum amyloid‐beta levels are increased in patients with obstructive sleep apnea syndrome. Scientific Reports, 5, 13917. 10.1038/srep13917 26351108 PMC4563592

[brb33306-bib-0010] Derry, C. , & Duncan, S. (2013). Sleep and epilepsy. Epilepsy & Behavior, 26(3), 394–404. 10.1016/j.yebeh.2012.10.033 23465654

[brb33306-bib-0011] Di Gennaro, G. , Quarato, P. P. , Onorati, P. , Colazza, G. B. , Mari, F. , Grammaldo, L. G. , Ciccarelli, O. , Meldolesi, N. G. , Sebastiano, F. , Manfredi, M. , & Esposito, V. (2003). Localizing significance of temporal intermittent rhythmic delta activity (TIRDA) in drug‐resistant focal epilepsy. Clinical Neurophysiology, 114(1), 70–78. 10.1016/s1388-2457(02)00332-2 12495766

[brb33306-bib-0012] D'Rozario, A. L. , Chapman, J. L. , Phillips, C. L. , Palmer, J. R. , Hoyos, C. M. , Mowszowski, L. , Duffy, S. L. , Marshall, N. S. , Benca, R. , Mander, B. , Grunstein, R. R. , & Naismith, S. L. (2020). Objective measurement of sleep in mild cognitive impairment: A systematic review and meta‐analysis. Sleep Medicine Reviews, 52, 101308. 10.1016/j.smrv.2020.101308 32302775

[brb33306-bib-0013] Emamian, F. , Khazaie, H. , Tahmasian, M. , Leschziner, G. D. , Morrell, M. J. , Hsiung, G.‐Y. R. , Rosenzweig, I. , & Sepehry, A. A. (2016). The association between obstructive sleep apnea and Alzheimer's disease: A meta‐analysis perspective. Frontiers in Aging Neuroscience, 8, 78. 10.3389/fnagi.2016.00078 27148046 PMC4828426

[brb33306-bib-0014] Ferman, T. J. , Smith, G. E. , Boeve, B. F. , Ivnik, R. J. , Petersen, R. C. , Knopman, D. , Graff‐Radford, N. , Parisi, J. , & Dickson, D. W. (2004). DLB fluctuations: Specific features that reliably differentiate DLB from AD and normal aging. Neurology, 62(2), 181–187. 10.1212/wnl.62.2.181 14745051

[brb33306-bib-0015] Folstein, M. F. , Folstein, S. E. , & Mchugh, P. R. (1975). Mini‐mental state. A practical method for grading the cognitive state of patients for the clinician. Journal of Psychiatric Research, 12(3), 189–198. 10.1016/0022-3956(75)90026-6 1202204

[brb33306-bib-0016] Friedman, D. , Honig, L. S. , & Scarmeas, N. (2012). Seizures and epilepsy in Alzheimer's disease. CNS Neuroscience & Therapeutics, 18(4), 285–294. 10.1111/j.1755-5949.2011.00251.x 22070283 PMC3630499

[brb33306-bib-0017] Gambardella, A. , Gotman, J. , Cendes, F. , & Andermann, F. (1995). Focal intermittent delta activity in patients with mesiotemporal atrophy: A reliable marker of the epileptogenic focus. Epilepsia, 36(2), 122–129. 10.1111/j.1528-1157.1995.tb00970.x 7821268

[brb33306-bib-0018] Gordon, N. P. , Yao, J. H. , Brickner, L. A. , & Lo, J. C. (2022). Prevalence of sleep‐related problems and risks in a community‐dwelling older adult population: A cross‐sectional survey‐based study. BMC Public Health [Electronic Resource], 22(1), 2045. 10.1186/s12889-022-14443-8 36348296 PMC9644466

[brb33306-bib-0019] Harper, R. M. , Poe, G.R., Rector, D. M. , & Kristensen, M. P. (1998). Relationships between hippocampal activity and breathing patterns. Neuroscience & Biobehavioral Reviews, 22(2), 233–236. 10.1016/s0149-7634(97)00010-9 9579314

[brb33306-bib-0020] Hirsch, L. J. , Fong, M. W. K. , Leitinger, M. , Laroche, S. M. , Beniczky, S. , Abend, N. S. , Lee, J. W. , Wusthoff, C. J. , Hahn, C. D. , Westover, M. B. , Gerard, E. E. , Herman, S. T. , Haider, H. A. , Osman, G. , Rodriguez‐Ruiz, A. , Maciel, C. B. , Gilmore, E. J. , Fernandez, A. , Rosenthal, E. S. , … Gaspard, N. (2021). American clinical neurophysiology society's standardized critical care EEG terminology: 2021 Version. Journal of Clinical Neurophysiology, 38(1), 1–29. 10.1097/WNP.0000000000000806 33475321 PMC8135051

[brb33306-bib-0021] Hoch, C. C. , Reynolds, C. F., 3rd , Kupfer, D. J. , Houck, P. R. , Berman, S. R. , & Stack, J. A. (1986). Sleep‐disordered breathing in normal and pathologic aging. Journal of Clinical Psychiatry, 47(10), 499–503. 3759914

[brb33306-bib-0022] Holth, J. K. , Fritschi, S. K. , Wang, C. , Pedersen, N. P. , Cirrito, J. R. , Mahan, T. E. , Finn, M. B. , Manis, M. , Geerling, J. C. , Fuller, P. M. , Lucey, B. P. , & Holtzman, D. M. (2019). The sleep‐wake cycle regulates brain interstitial fluid tau in mice and CSF tau in humans. Science, 363(6429), 880–884. 10.1126/science.aav2546 30679382 PMC6410369

[brb33306-bib-0023] Holth, J. K. , Patel, T. K. , & Holtzman, D. M. (2017). Sleep in Alzheimer's disease—Beyond amyloid. Neurobiology of Sleep and Circadian Rhythms, 2, 4–14. 10.1016/j.nbscr.2016.08.002 28217760 PMC5312809

[brb33306-bib-0024] Horváth, A. , Szűcs, A. , Barcs, G. , & Kamondi, A. (2017). Sleep EEG detects epileptiform activity in Alzheimer's disease with high sensitivity. Journal of Alzheimer's Disease, 56(3), 1175–1183. 10.3233/JAD-160994 28128769

[brb33306-bib-0025] Horvath, A. A. , Papp, A. , Zsuffa, J. , Szucs, A. , Luckl, J. , Radai, F. , Nagy, F. , Hidasi, Z. , Csukly, G. , Barcs, G. , & Kamondi, A. (2021). Subclinical epileptiform activity accelerates the progression of Alzheimer's disease: A long‐term EEG study. Clinical Neurophysiology, 132(8), 1982–1989. 10.1016/j.clinph.2021.03.050 34034963

[brb33306-bib-0026] Johns, M. W. (1991). A new method for measuring daytime sleepiness: The Epworth sleepiness scale. Sleep, 14(6), 540–545. 10.1093/sleep/14.6.540 1798888

[brb33306-bib-0027] Ju, Y. E. , Finn, M. B. , Sutphen, C. L. , Herries, E. M. , Jerome, G. M. , Ladenson, J. H. , Crimmins, D. L. , Fagan, A. M. , & Holtzman, D. M. (2016). Obstructive sleep apnea decreases central nervous system‐derived proteins in the cerebrospinal fluid. Annals of Neurology, 80(1), 154–159. 10.1002/ana.24672 27129429 PMC5120585

[brb33306-bib-0028] Juva, K. , Sulkava, R. , Erkinjuntti, T. , Ylikoski, R. , Valvanne, J. , & Tilvis, R. (1995). Usefulness of the clinical dementia rating scale in screening for dementia. International Psychogeriatrics, 7(1), 17–24. 10.1017/s1041610295001815 7579017

[brb33306-bib-0029] Kural, M. A. , Duez, L. , Sejer Hansen, V. , Larsson, P. G. , Rampp, S. , Schulz, R. , Tankisi, H. , Wennberg, R. , Bibby, B. M. , Scherg, M. , & Beniczky, S. , Sejer Hansen, V. (2020). Criteria for defining interictal epileptiform discharges in EEG: A clinical validation study. Neurology, 94(20), e2139–e2147. 10.1212/WNL.0000000000009439 32321764 PMC7526669

[brb33306-bib-0030] Lam, A. D. , Deck, G. , Goldman, A. , Eskandar, E. N. , Noebels, J. , & Cole, A. J. (2017). Silent hippocampal seizures and spikes identified by foramen ovale electrodes in Alzheimer's disease. Nature Medicine, 23(6), 678–680. 10.1038/nm.4330 PMC5461182 28459436

[brb33306-bib-0031] Lam, A. D. , Sarkis, R. A. , Pellerin, K. R. , Jing, J. , Dworetzky, B. A. , Hoch, D. B. , Jacobs, C. S. , Lee, J. W. , Weisholtz, D. S. , Zepeda, R. , Westover, M. B. , Cole, A. J. , & Cash, S. S. (2020). Association of epileptiform abnormalities and seizures in Alzheimer disease. Neurology, 95(16), e2259–e2270. 10.1212/WNL.0000000000010612 32764101 PMC7713786

[brb33306-bib-0032] Lam, A. D. , Zepeda, R. , Cole, A. J. , & Cash, S. S. (2016). Widespread changes in network activity allow non‐invasive detection of mesial temporal lobe seizures. Brain, 139(Pt 10), 2679–2693. 10.1093/brain/aww198 27474219 PMC5035820

[brb33306-bib-0033] Lieb, J. P. , Walsh, G. O. , Babb, T. L. , Walter, R. D. , Crandall, P. H. , Tassinari, C. A. , Portera, A. , & Scheffner, D. (1976). A comparison of EEG seizure patterns recorded with surface and depth electrodes in patients with temporal lobe epilepsy. Epilepsia, 17(2), 137–160. 10.1111/j.1528-1157.1976.tb03392.x 947745

[brb33306-bib-0034] Liguori, C. , Cremascoli, R. , Maestri, M. , Fernandes, M. , Izzi, F. , Tognoni, G. , Scarpina, F. , Siciliano, G. , Mercuri, N. B. , Priano, L. , Bonanni, E. , & Placidi, F. (2021). Obstructive sleep apnea syndrome and Alzheimer's disease pathology: May continuous positive airway pressure treatment delay cognitive deterioration?, Sleep and Breathing, 25(4), 2135–2139. 10.1007/s11325-021-02320-4 33619666

[brb33306-bib-0035] Liguori, C. , Mercuri, N. B. , Izzi, F. , Romigi, A. , Cordella, A. , Sancesario, G. , & Placidi, F. (2017). Obstructive sleep apnea is associated with early but possibly modifiable Alzheimer's disease biomarkers changes. Sleep, 40(5), 10.1093/sleep/zsx011 28329084

[brb33306-bib-0036] Liguori, C. , Mercuri, N. B. , Nuccetelli, M. , Izzi, F. , Cordella, A. , Bernardini, S. , & Placidi, F. (2019). Obstructive sleep apnea may induce orexinergic system and cerebral beta‐amyloid metabolism dysregulation: Is it a further proof for Alzheimer's disease risk? Sleep Medicine, 56, 171–176. 10.1016/j.sleep.2019.01.003 30799255

[brb33306-bib-0037] Lin, Z. , Si, Q. I. , & Xiaoyi, Z. (2017). Obstructive sleep apnoea in patients with epilepsy: A meta‐analysis. Sleep & Breathing = Schlaf & Atmung, 21(2), 263–270. 10.1007/s11325-016-1391-3 27473532

[brb33306-bib-0038] Lucey, B. P. , Wisch, J. , Boerwinkle, A. H. , Landsness, E. C. , Toedebusch, C. D. , Mcleland, J. S. , Butt, O. H. , Hassenstab, J. , Morris, J. C. , Ances, B. M. , & Holtzman, D. M. (2021). Sleep and longitudinal cognitive performance in preclinical and early symptomatic Alzheimer's disease. Brain, 144(9), 2852–2862. 10.1093/brain/awab272 34668959 PMC8536939

[brb33306-bib-0039] Manni, R. , & Terzaghi, M. (2010). Comorbidity between epilepsy and sleep disorders. Epilepsy Research, 90(3), 171–177. 10.1016/j.eplepsyres.2010.05.006 20570109

[brb33306-bib-0040] Matos, G. , Tufik, S. , Scorza, F. A. , Cavalheiro, E. A. , & Andersen, M. L. (2013). Sleep and epilepsy: Exploring an intriguing relationship with a translational approach. Epilepsy & Behavior, 26(3), 405–409. 10.1016/j.yebeh.2012.12.003 23394796

[brb33306-bib-0041] Mckhann, G. M. , Knopman, D. S. , Chertkow, H. , Hyman, B. T. , Jack, C. R. , Kawas, C. H. , Klunk, W. E. , Koroshetz, W. J. , Manly, J. J. , Mayeux, R. , Mohs, R. C. , Morris, J. C. , Rossor, M. N. , Scheltens, P. , Carrillo, M. C. , Thies, B. , Weintraub, S. , & Phelps, C. H. (2011). The diagnosis of dementia due to Alzheimer's disease: Recommendations from the National Institute on Aging‐Alzheimer's Association workgroups on diagnostic guidelines for Alzheimer's disease. Alzheimer's & Dementia, 7(3), 263–269. 10.1016/j.jalz.2011.03.005 PMC3312024 21514250

[brb33306-bib-0042] Miller, D. J. , Sargent, C. , & Roach, G. D. (2022). A validation of six wearable devices for estimating sleep, heart rate and heart rate variability in healthy adults. Sensors, 22(16), 6317. 10.3390/s22166317 36016077 PMC9412437

[brb33306-bib-0043] Mollayeva, T. , Thurairajah, P. , Burton, K. , Mollayeva, S. , Shapiro, C. M. , & Colantonio, A. (2016). The Pittsburgh sleep quality index as a screening tool for sleep dysfunction in clinical and non‐clinical samples: A systematic review and meta‐analysis. Sleep Medicine Reviews, 25, 52–73. 10.1016/j.smrv.2015.01.009 26163057

[brb33306-bib-0044] Motamedi, V. , Kanefsky, R. , Matsangas, P. , Mithani, S. , Jeromin, A. , Brock, M. S. , Mysliwiec, V. , & Gill, J. (2018). Elevated tau and interleukin‐6 concentrations in adults with obstructive sleep apnea. Sleep Medicine, 43, 71–76. 10.1016/j.sleep.2017.11.1121 29482817

[brb33306-bib-0045] Nobili, L. , Frauscher, B. , Eriksson, S. , Gibbs, S. A. , Halasz, P. , Lambert, I. , Manni, R. , Peter‐Derex, L. , Proserpio, P. , Provini, F. , De Weerd, A. , & Parrino, L. (2022). Sleep and epilepsy: A snapshot of knowledge and future research lines. Journal of Sleep Research, 31, e13622. 10.1111/jsr.13622 35487880 PMC9540671

[brb33306-bib-0046] Normand, M. M. , Wszolek, Z. K. , & Klass, D. W. (1995). Temporal intermittent rhythmic delta activity in electroencephalograms. Journal of Clinical Neurophysiology, 12(3), 280–284. 10.1097/00004691-199505010-00005 11221786

[brb33306-bib-0047] Osorio, R. S. , Ayappa, I. , Mantua, J. , Gumb, T. , Varga, A. , Mooney, A. M. , Burschtin, O. E. , Taxin, Z. , During, E. , Spector, N. , Biagioni, M. , Pirraglia, E. , Lau, H. , Zetterberg, H. , Blennow, K. , Lu, S.‐E. , Mosconi, L. , Glodzik, L. , Rapoport, D. M. , & De Leon, M. J. (2014). The interaction between sleep‐disordered breathing and apolipoprotein E genotype on cerebrospinal fluid biomarkers for Alzheimer's disease in cognitively normal elderly individuals. Neurobiology of Aging, 35(6), 1318–1324. 10.1016/j.neurobiolaging.2013.12.030 24439479 PMC4022140

[brb33306-bib-0048] Osorio, R. S. , Gumb, T. , Pirraglia, E. , Varga, A. W. , Lu, S.‐E. , Lim, J. , Wohlleber, M. E. , Ducca, E. L. , Koushyk, V. , Glodzik, L. , Mosconi, L. , Ayappa, I. , Rapoport, D. M. , De Leon, M. J. , Weiner, M. W. , Aisen, P. , Petersen, R. , Jack, C. , Jagust, W. , … Beckett, L. (2015). Sleep‐disordered breathing advances cognitive decline in the elderly. Neurology, 84(19), 1964–1971. 10.1212/wnl.0000000000001566 25878183 PMC4433459

[brb33306-bib-0049] Park, S.‐H. , & Cho, Y. S. (2022). Predictive validity of the Cornell Scale for depression in dementia among older adults with and without dementia: A systematic review and meta‐analysis. Psychiatry Research, 310, 114445. 10.1016/j.psychres.2022.114445 35190341

[brb33306-bib-0050] Peter‐Derex, L. , Klimes, P. , Latreille, V. , Bouhadoun, S. , Dubeau, F. , & Frauscher, B. (2020). Sleep disruption in epilepsy: Ictal and interictal epileptic activity matter. Annals of Neurology, 88(5), 907–920. 10.1002/ana.25884 32833279

[brb33306-bib-0051] Peter‐Derex, L. , Yammine, P. , Bastuji, H. , & Croisile, B. (2015). Sleep and Alzheimer's disease. Sleep Medicine Reviews, 19, 29–38. 10.1016/j.smrv.2014.03.007 24846773

[brb33306-bib-0052] Pornsriniyom, D. , Kim, H. W. , Bena, J. , Andrews, N. D. , Moul, D. , & Foldvary‐Schaefer, N. (2014). Effect of positive airway pressure therapy on seizure control in patients with epilepsy and obstructive sleep apnea. Epilepsy & Behavior, 37, 270–275. 10.1016/j.yebeh.2014.07.005 25117208

[brb33306-bib-0053] Pornsriniyom, D. , Shinlapawittayatorn, K. , Fong, J. , Andrews, N. D. , & Foldvary‐Schaefer, N. (2014). Continuous positive airway pressure therapy for obstructive sleep apnea reduces interictal epileptiform discharges in adults with epilepsy. Epilepsy & Behavior, 37, 171–174. 10.1016/j.yebeh.2014.06.025 25042599

[brb33306-bib-0054] Reiher, J. , Beaudry, M. , & Leduc, C. P. (1989). Temporal intermittent rhythmic delta activity (TIRDA) in the diagnosis of complex partial epilepsy: Sensitivity. specificity and predictive value. Canadian Journal of Neurological Sciences, 16(4), 398–401. 10.1017/s0317167100029450 2804800

[brb33306-bib-0055] Reutens, D. C. , Howell, R. A. , Gebert, K. E. , & Berkovic, S. F. (1992). Validation of a questionnaire for clinical seizure diagnosis. Epilepsia, 33(6), 1065–1071. 10.1111/j.1528-1157.1992.tb01760.x 1464265

[brb33306-bib-0056] Reynolds, C. F., 3rd , Kupfer, D. J. , & Taska, L. S. (1985). Sleep apnea in Alzheimer's dementia: Correlation with mental deterioration. Journal of Clinical Psychiatry, 46(7), 257–261. 4008448

[brb33306-bib-0057] Romanella, S. M. , Roe, D. , Tatti, E. , Cappon, D. , Paciorek, R. , Testani, E. , Rossi, A. , Rossi, S. , & Santarnecchi, E. (2021). The sleep side of aging and Alzheimer's disease. Sleep Medicine, 77, 209–225. 10.1016/j.sleep.2020.05.029 32912799 PMC8364256

[brb33306-bib-0058] Sadak, U. , Honrath, P. , Ermis, U. , Heckelmann, J. , Meyer, T. , Weber, Y. , & Wolking, S. (2022). Reduced REM sleep: A potential biomarker for epilepsy—A retrospective case‐control study. Seizure: The Journal of the British Epilepsy Association, 98, 27–33. 10.1016/j.seizure.2022.03.022 35398671

[brb33306-bib-0059] Scharf, M. T. (2022). Reliability and efficacy of the epworth sleepiness scale: Is there still a place for it? Nature and science of sleep, 14, 2151–2156. 10.2147/NSS.S340950 PMC9759004 36536636

[brb33306-bib-0060] Schiller, K. , Avigdor, T. , Abdallah, C. , Sziklas, V. , Crane, J. , Stefani, A. , Peter‐Derex, L. , & Frauscher, B. (2022). Focal epilepsy disrupts spindle structure and function. Scientific Reports, 12(1), 11137. 10.1038/s41598-022-15147-0 35778434 PMC9249850

[brb33306-bib-0061] Spira, A. P. , Yager, C. , Brandt, J. , Smith, G. S. , Zhou, Y. , Mathur, A. , Kumar, A. , Brašić, J. R. , Wong, D. F. , & Wu, M. N. (2014). Objectively measured sleep and beta‐amyloid burden in older adults: a pilot study. SAGE Open Medicine, 2, 2050312114546520. 10.1177/2050312114546520 25621174 PMC4304392

[brb33306-bib-0062] Troussière, A.‐C. , Monaca Charley, C. , Salleron, J. , Richard, F. , Delbeuck, X. , Derambure, P. , Pasquier, F. , & Bombois, S. (2014). Treatment of sleep apnoea syndrome decreases cognitive decline in patients with Alzheimer's disease. Journal of Neurology, Neurosurgery, and Psychiatry, 85(12), 1405–1408. 10.1136/jnnp-2013-307544 24828897

[brb33306-bib-0063] Van Golde, E. G. A. , Gutter, T. , & De Weerd, A. W. (2011). Sleep disturbances in people with epilepsy; prevalence, impact and treatment. Sleep Medicine Reviews, 15(6), 357–368. 10.1016/j.smrv.2011.01.002 21439869

[brb33306-bib-0064] Vossel, K. , Ranasinghe, K. G. , Beagle, A. J. , La, A. , Ah Pook, K. , Castro, M. , Mizuiri, D. , Honma, S. M. , Venkateswaran, N. , Koestler, M. , Zhang, W. , Mucke, L. , Howell, M. J. , Possin, K. L. , Kramer, J. H. , Boxer, A. L. , Miller, B. L. , Nagarajan, S. S. , & Kirsch, H. E. (2021). Effect of levetiracetam on cognition in patients with Alzheimer disease with and without epileptiform activity: A randomized clinical trial. JAMA Neurology, 78(11), 1345–1354. 10.1001/jamaneurol.2021.3310 34570177 PMC8477304

[brb33306-bib-0065] Vossel, K. A. , Beagle, A. J. , Rabinovici, G. D. , Shu, H. , Lee, S. E. , Naasan, G. , Hegde, M. , Cornes, S. B. , Henry, M. L. , Nelson, A. B. , Seeley, W. W. , Geschwind, M. D. , Gorno‐Tempini, M. L. , Shih, T. , Kirsch, H. E. , Garcia, P. A. , Miller, B. L. , & Mucke, L. (2013). Seizures and epileptiform activity in the early stages of Alzheimer disease. JAMA Neurology, 70(9), 1158–1166. 10.1001/jamaneurol.2013.136 23835471 PMC4013391

[brb33306-bib-0066] Vossel, K. A. , Ranasinghe, K. G. , Beagle, A. J. , Mizuiri, D. , Honma, S. M. , Dowling, A. F. , Darwish, S. M. , Van Berlo, V. , Barnes, D. E. , Mantle, M. , Karydas, A. M. , Coppola, G. , Roberson, E. D. , Miller, B. L. , Garcia, P. A. , Kirsch, H. E. , Mucke, L. , & Nagarajan, S. S. (2016). Incidence and impact of subclinical epileptiform activity in Alzheimer's disease. Annals of Neurology, 80(6), 858–870. 10.1002/ana.24794 27696483 PMC5177487

[brb33306-bib-0067] Vossel, K. A. , Tartaglia, M. C. , Nygaard, H. B. , Zeman, A. Z. , & Miller, B. L. (2017). Epileptic activity in Alzheimer's disease causes and clinical relevance. The Lancet, 16, 311–322. 10.1016/S1474-4422(17)30044-3 28327340 PMC5973551

[brb33306-bib-0068] Yeh, W.‐C. , Lai, C.‐L. , Wu, M.‐N. , Lin, H.‐C. , Lee, K.‐W. , Li, Y.‐S. , & Hsu, C.‐Y. (2021). Rapid eye movement sleep disturbance in patients with refractory epilepsy: A polysomnographic study. Sleep Medicine, 81, 101–108. 10.1016/j.sleep.2021.02.007 33647761

[brb33306-bib-0069] Yeh, W.‐C. , Lin, H.‐J. , Li, Y.‐S. , Chien, C.‐F. , Wu, M.‐N. , Liou, L.‐M. , Hsieh, C.‐F. , & Hsu, C.‐Y. (2022). Rapid eye movement sleep reduction in patients with epilepsy: A systematic review and meta‐analysis. Seizure: The Journal of the British Epilepsy Association, 96, 46–58. 10.1016/j.seizure.2022.01.014 35123233

[brb33306-bib-0070] Zhang, Y. , Ren, R. , Yang, L. , Zhang, H. , Shi, Y. , Okhravi, H. R. , Vitiello, M. V. , Sanford, L. D. , & Tang, X. (2022). Sleep in Alzheimer's disease: A systematic review and meta‐analysis of polysomnographic findings. Translational Psychiatry, 12(1), 136. 10.1038/s41398-022-01897-y 35365609 PMC8976015

