# Supplementary material for: Subclinical epileptiform activity and sleep disturbances in Alzheimer's disease
Source: Brain Behav. 2023 Nov 10;13(12):e3306. doi: 10.1002/brb3.3306 (PMC10726840; doi:10.1002/brb3.3306)
Supplement: Supplementary file 1 — APPENDIX A: BIOMARKER DATA AND DISEASE STAGE OF AD PATIENTS APPENDIX B: CLINICAL AND DEMOGRAPHICAL PARAMETERS AD‐EPI+ VERSUS AD‐EPI– SUBGROUP [file BRB3-13-e3306-s001.docx]

Appendix A: Biomarker data and disease stage of AD patients

| **Patient Nr** | **Disease stage** | **Amyloïd-PET** | **CSF Biomarkers** | **^18^F- FDG-PET** | **Brain MRI (or CT)** | **ApoE** |
| --- | --- | --- | --- | --- | --- | --- |
| 1 | AD | NA | NA | NA | Generalized atrophy | NA |
| 2 | AD | NA | NA | NA | CT: Generalized atrophy, frontotemporal predominance | NA |
| 3 | AD | NA | NA | NA | Generalized atrophy. Prominent mesial temporal atrophy | NA |
| 4 | AD | NA | NA | NA | CT: Generalized atrophy | E3/E4 |
| 5 | AD | NA | t-tau = 365  p-tau 181 = 52.7  Aβ 1-42 = 501^a^ | Hypometabolism temporoparietal, precuneus and frontal | Normal for age | NA |
| 6 | AD | NA | NA | NA | Generalized atrophy. Mild mesial temporal and parietal atrophy | E3/E3 |
| 7 | naMCI | Positive | Aβ 1-42 = 442 Ratio 1-42/1-40 = 0.087 p-tau = 90.4 t-tau = 592^b^ | Mild hypometabolism temporal and parietal bilateral | Normal for age | E4/E4 |
| 8 | AD | NA | NA | NA | Generalized atrophy. Prominent mesial temporal atrophy. | E3/E3 |
| 9 | AD | NA | NA | NA | Generalized atrophy, frontotemporal predominance. Prominent mesial temporal atrophy R > L | NA |
| 10 | AD | NA | NA | NA | Generalized atrophy, parietal predominance. Prominent mesial temporal atrophy R > L | NA |
| 11 | naMCI | NA | Ratio 1-42/1-40 = 0.076  Aβ 1-42 = 410  t-tau = 275^c^ | No hypometabolism | Mild generalized atrophy. Moderate vascular white matter lesions. | E3/E4 |
| 12 | AD | NA | t-tau = 581  p-tau = 80.9 Aβ 1-42 = 695^a^ | Hypometabolism frontal R, bilateral parietal and cuneus R > L | Mild generalized atrophy. Mild vascular white matter lesions. FLAIR hyperintensity frontal right, parietal left and insula (old ischemic lesions). | NA |
| 13 | AD | NA | t-tau = 770 p-tau = 107.8  Aβ 1-42 = 332^a^ | Hypometabolism temporoparietal R > L, precuneus and frontal L | Generalized atrophy, parietal and temporal predominance. Prominent mesial temporal atrophy R > L | NA |
| 14 | naMCI | NA | Aβ 1-42 = 452  Aβ 1-40 = 6765  Ratio 1-42/1-40 = 0.067 t-tau = 425  p-tau = 58^c^ | NA | Frontoparietal atrophy. Mild mesial temporal atrophy | NA |
| 15 | AD | NA | NA | NA | Prominent temporoparietal atrophy. Prominent mesial temporal atrophy L > R | E3/E4 |
| 16 | AD | Positive | NA | NA | Normal for age | NA |
| 17 | AD | NA | NA | NA | Generalized atrophy, parietal predominance. Prominent mesial temporal atrophy L > R. Mild to moderate white matter lesions. | NA |
| 18 | aMCI | NA | NA | NA | Generalized atrophy. Prominent mesial temporal atrophy R > L. Prominent white matter lesions. | E3/E4 |
| 19 | naMCI | Positive | NA | Hypometabolism temporoparietal L > R, frontal L, precuneus L > R | Parietal atrophy. Mild white matter lesions. | E4/E4 |
| 20 | AD | NA | NA | Mild hypometabolism frontal L | Mild generalized atrophy. Mild to moderate white matter lesions. Old lacunar infarct thalamus L | NA |
| 21 | AD | NA | NA | Hypometabolism bilateral parietal and mild temporoparietal L | Generalized atrophy, temporoparietal predominance. | NA |
| 22 | AD | NA | NA | Hypometabolism parietotemporal L > R, frontal L and precuneus bilateral | Mild generalized atrophy. Symmetric temporal lobe atrophy. | NA |
| 23 | aMCI | NA | Aβ 1-42 = 361 Aβ 1-40 = 7026 Ratio 1-42/1-40 = 0.051 t-tau = 715 p-tau = 110^c^ | NA | Generalized atrophy. Prominent mesial temporal atrophy. Moderate vascular white matter lesions. Gliosis frontoparietal right. | NA |
| 24 | AD | NA | NA | NA | Prominent mesial temporal atrophy L > R. Periventricular leukoencephalopathy | E2/E4 |
| 25 | AD | NA | Aβ 1-42 = 388  Aβ 1-40 = 7588  Ratio 1-42/1-40 = 0.051  t-tau = 362^c^ | NA | Generalized atrophy, frontoparietal predominance. Prominent mesial temporal atrophy. Mild to moderate white matter lesions. | NA |
| 26 | naMCI | NA | NA | NA | CT: Mild parietal atrophy. | NA |
| 27 | AD | NA | NA | Hypometabolism temporoparietal R > L, precuneus and posterior cingulate bilateral | Generalized atrophy. Moderate mesial temporal atrophy. Mild to moderate white matter lesions. | NA |
| 28 | AD | NA | NA | NA | Generalized atrophy. Moderate mesial temporal atrophy. Mild to moderate periventricular white matter lesions. | NA |
| 29 | AD | NA | Aβ 1-42 = 309  Aβ 1-40 = 7663  Ratio 1-42/1-40 = 0.040  t-tau = 1034^c^ | NA | Generalized atrophy, frontoparietal predominance. Prominent mesial temporal atrophy. Mild to moderate white matter lesions. | E3/E3 |
| 30 | AD | NA | NA | NA | Generalized atrophy. Prominent mesial temporal atrophy L > R. Mild to moderate white matter lesions. | NA |
| 31 | aMCI | NA | Aβ 1-42 = 667  Aβ 1-40 = 8838  Ratio 1-42/1-40 = 0.075  t-tau = 674^c^ | Hypometabolism parietotemporal bilateral, frontal bilateral, precuneus and posterior cingulate. | Mild generalized atrophy. Prominent parietal atrophy. Mild mesial temporal atrophy L > R. Mild white matter lesions. | NA |
| 32 | aMCI | NA | Aβ 1-42 = 362  Aβ 1-40 = 5050 Ratio 1-42/1-40 = 0.072 t-tau = 314^c^ | Mild hypometabolism precuneus and posterior cingulate bilateral. | Mild mesial temporal atrophy | E3/E4 |
| 33 | aMCI | NA | NA | NA | Generalized atrophy. Prominent mesial temporal atrophy L > R. Mild white matter lesions. | NA |
| 34 | AD | NA | NA | Hypometabolism temporoparietal L > R, precuneus bilateral | Generalized atrophy, parietal predominance. Mesial temporal atrophy L > R. Mild white matter lesions | NA |
| 35 | AD | NA | Aβ 1-42 = 629  Aβ 1-40 = 10246  Ratio 1-42/1-40 = 0.061  t-tau = 526^c^ | Hypometabolism temporoparietal L > R and mild frontal L | CT: No significant changes | NA |
| 36 | AD | Positive | NA | Hypometabolism temporoparietal L > R, frontal L > R, precuneus and posterior cingulate, secondary visual cortex L | Generalized atrophy. Hemosiderin depositions cortico-subcortical. Mild to moderate white matter lesions | NA |
| 37 | aMCI | NA | Aβ 1-42 = 370 Aβ 1-40 = 6088  Ratio 1-42/1-40 = 0.061  t-tau = 389^c^ | Mild hypometabolism precuneus | Generalized atrophy, parieto-occipital predominance. Prominent mesial temporal atrophy. Prominent white matter lesions. | NA |
| 38 | AD | NA | NA | NA | Mild generalized atrophy. Mild mesial temporal atrophy. | E3/E4 |
| 39 | AD | Positive | NA | Hypometabolism parieto-temporal L > R, precuneus and posterior cingulate bilateral | Mild generalized atrophy. Mild white matter lesions. | E3/E3 |
| 40 | AD | NA | Aβ 1-42 = 482  Aβ 1-40 = 9886  Ratio 1-42/1-40 = 0.049 t-tau = 880^c^ | NA | Generalized atrophy. Prominent parietal atrophy. Prominent mesial temporal atrophy. | NA |
| 41 | aMCI | NA | Aβ 1-42 = 439  Aβ 1-40 = 9506  Ratio 1-42/1-40 = 0.046  t-tau = 1173^c^ | Mild hypometabolism parietotemporal, bilateral precuneus and posterior cingulate bilateral | Mild generalized atrophy. Mild parietal atrophy. Mild mesial temporal atrophy. | NA |

*^a^ Values supporting a diagnosis of Alzheimer’s disease are t-tau level > 367 pg/mL, p-tau > 80 pg/mL, Aβ 1-42 < 500 pg/mL*

*^b^ Values supporting a diagnosis of Alzheimer’s disease are p-tau > 57pg/mL, t-tau > 501 pg/mL, ratio 1-42/1-40 < 0.12, Aβ 1-42 < 775 pg/mL*

*^c^ Values supporting a diagnosis of Alzheimer’s disease are ratio 1-42/1-40 < 0.096, t-tau > 545 pg/mL*

*Amyloid PET was performed with ^11^C-Pittsburgh compound B, except for subj 36 with ^18^F-NAV4694*

*Aβ 1-40 = amyloid-beta peptide ending in amino acid residue 40; Aβ 1-42 = amyloid-beta peptide ending in amino acid residue 42; aMCI = Amnestic mild cognitive impairment; ApoE = Apolipoprotein E; CSF = Cerebrospinal fluid; CT = computed tomography; L = left; MRI = magnetic resonance imaging; NA = Not Applicable; naMCI = non-amnestic mild cognitive impairment; PET = Positron emission tomography; p-tau = tau phosphorylated at threonine 181; R = right; t-tau = total tau; ^18^F-FDG-PET = [^18^F]-fluoro-2-deoxyglucose*

Appendix B: Clinical and demographical parameters AD-Epi+ vs. AD-Epi- subgroup

|  | AD-Epi+ (n = 14) | AD-Epi- (n = 27) | p-value |
| --- | --- | --- | --- |
| Number of male (%) | 7 (50%) | 15 (56%) | N.S. |
| Number of participants with MCI (%) | 6 (43%) | 6 (22%) | N.S. |
| Median duration of symptoms in years [IQR] | 3 [2 - 5] | 4 [3 – 6] | N.S. |
| Mean age in years (SD) | 73 (7) | 76 (6) | 0.077 |
| Mean BMI in kg/m^2^ (SD) | 24.80 (1.87) | 22.71 (3.85) | 0.024 * |
| Median units ethyl/day [IQR] | 0 [0 – 0] | 1 [0 – 2] | N.S. |
| Median units caffeine consumption day [IQR] | 3 [2 – 5] | 3 [2 – 5] | N.S. |
| Number of participants who smoke (%) | 2 (14%) | 3 (11%) | N.S. |
| Number of participants who took benzodiazepine, anti-depressive or anti-psychotic drug (%) | 5 (36%) | 11 (41%) | N.S. |
| Median MMSE [IQR] | 24 [16 – 28] | 20 [15 – 24] | N.S. |
| Median CDR [IQR] | 1 [0.5 – 2.0] | 1 [0.5 – 2.0] | N.S. |
| Median ESS [IQR] | 5 [1 - 9] | 5 [2 – 8] | N.S. |
| Median PSQI [IQR] | 2 [2 – 4] | 3 [2 – 6] | N.S. |
| Median Cornell [IQR] | 4 [1 – 6] | 7 [2 – 11] | 0.050 * |
| Median Mayo [IQR] | 0 [0 – 1] | 1 [0 – 2] | N.S. |
| Number of participants with Positive Reutens Questionnaire (%) | 2 (15%) | 1 (14%) | N.S. |
| Number of participants with OSAS (%)^†^ | 10 (77%) | 18 (72%) | N.S. |

Abbreviations: *BMI = Body Mass Index; CDR = Clinical Dementia Rating Scale; ESS = Epworth Sleepiness Scale; IQR = Inter Quartile Range; MCI = Mild Cognitive Impairment; MMSE = Mini Mental State Examination; N.S. = Not significant; OSAS = Obstructive Sleep Apnea Syndrome; PSQI = Pittsburg Sleep Quality Index; SD = Standard Deviation*

Significant p-values ( ≤ 0.05) are marked with *

^†^ Based on dataset after excluding not reliable polysomnography’s (AD-Epi+ n = 13, AD-Epi- n = 15)
